# Overcoming the therapeutic plateau in overactive bladder: a grand challenge in female urology

**DOI:** 10.3389/fruro.2025.1654550

**Published:** 2025-08-12

**Authors:** Bilal Chughtai, Jennifer Polo, Naeem Bhojani, Kevin C. Zorn, Dean Elterman

**Affiliations:** ^1^ Department of Urology, Northwell Health, Plainview Hospital, New York, NY, United States; ^2^ Department of Urology, Donald and Barbara Zucker School of Medicine at Hofstra/Northwell, Hempstead, NY, United States; ^3^ Faculty of Medicine, Université de Montréal, Montreal, QC, Canada; ^4^ Division of Urology, Department of Surgery, Université de Montréal Health Center, Montreal, QC, Canada; ^5^ Benign Prostatic Hyperplasia (BPH) Canada Prostate Surgical Institute, Mont-Royal Surgical Center, Montreal, QC, Canada; ^6^ Division of Urology, Department of Surgery, University of Toronto, Toronto, ON, Canada

**Keywords:** overactive bladder (OAB), urinary urgency, female urology, therapeutic plateau, digital therapeutics, biomarkers, neuromodulation

## 1 Defining the challenge: what is overactive bladder?

Overactive bladder (OAB) is a symptom-based syndrome characterized by urinary urgency, usually accompanied by frequency and nocturia, with or without urgency urinary incontinence, in the absence of infection or other identifiable pathology. While disproportionately affecting women, OAB also occurs in men, often alongside conditions like benign prostatic hyperplasia (BPH). Recognizing sex- and gender-based differences in presentation and treatment response is essential for inclusive and effective care.

Despite its high prevalence - affecting 30–40% of women (1, 2) – OAB remain underdiagnosed, and undertreated. Common therapies such as antimuscarinics and beta-3 agonists suffer from limited adherence due to side effects and insurance hurdles (3). Invasive options like neuromodulation and botulinum toxin injections, although effective for some, after often reserved for late-stage intervention (4). The current model of care fails to address the chronic and multidimensional nature of the condition.

## 2 Biological, behavioral, and molecular complexity

OAB is not a single disease entity, but rather a multifactorial syndrome shaped by hormonal status, aging, pelvic floor integrity, neurologic input, and psychosocial stressors. This complexity is often overlooked in current clinical pathways, which remain overly linear and simplified. Diagnostic tools such as urodynamics are underutilized or misinterpreted, and behavioral components are rarely addressed in a structured way. Many patients present with mixed symptoms or comorbid pelvic pain, yet care remains fragmented.

Early diagnosis is foundational to effective OAB management but remains a significant gap in practice. Many women delay seeking care due to embarrassment or the misconception that symptoms are a normal part of aging. Primary care providers may miss subtle signs or misattribute symptoms to less concerning causes. Increasing awareness and equipping clinicians with accessible screening tools—such as symptom checklists, digital pre-screening apps, and validated questionnaires—could significantly reduce time to treatment, particularly among high-risk and postmenopausal women.

A recent community-based study by Chughtai et al. demonstrated that targeted education among minority women significantly improved OAB symptom control and quality of life, highlighting the impact of structured outreach and patient empowerment. Targeted education programs, especially in underserved communities, have demonstrated encouraging outcomes in symptom relief and quality of life. Early intervention is essential not only to improve patient well-being, but also to prevent progression and long-term complications.

In parallel, emerging molecular research is reshaping how OAB might be understood and stratified. Biomarkers associated with central and peripheral neural function, smooth muscle architecture, and extracellular matrix remodeling offer promise for more refined phenotyping and individualized therapy. Epigenetic mechanisms that regulate detrusor muscle activity and inflammation may also contribute to symptom development. Moving forward, interdisciplinary collaboration—including input from basic scientists, molecular biologists, and neurobiologists—will be essential to fully explore these pathways and translate them into meaningful clinical applications.

## 3 Diagnostic pathways and their limitations

Although multiple guideline-based treatment options are available, patient satisfaction remains low (3). Pharmacologic therapies, particularly oral medications, are often discontinued due to side effects, limited effectiveness, or insurance restrictions. More advanced options like percutaneous tibial nerve stimulation and sacral neuromodulation can be effective, but they require sustained time, access, and commitment—barriers that deter many patients. As a result, treatment is frequently discontinued after one or two unsuccessful attempts.

To improve outcomes, the treatment paradigm must prioritize persistence, personalization, and patient-centered approaches over rigid, stepwise algorithms (5).

On the diagnostic side, evaluations typically start with symptom questionnaires, voiding diaries, and urinalysis. Urodynamic studies, while informative, are often underutilized due to their invasive nature, cost, and limited availability. Imaging is generally reserved for complicated cases. These diagnostic tools are not consistently integrated into a cohesive framework and often fail to account for the complex and multifactorial nature of OAB.

There is a clear need for a more comprehensive, non-invasive diagnostic model that incorporates behavioral assessments, validated instruments, wearable sensor data, and potentially molecular diagnostics. Such a framework could improve diagnostic accuracy, personalize treatment plans, and shorten the interval between symptom onset and effective intervention.

## 4 Therapeutic plateau and underlying barriers

While guideline-based treatments exist, patient satisfaction remains low due to fragmented care and poor follow-through. Real-world adherence to medications is limited by tolerability and systemic barriers. Even advanced therapies such as sacral neuromodulation or tibial nerve stimulation are underutilized due to logistical challenges (6).

Emerging technologies—such as app-based bladder training, AI-assisted voiding diaries, digital behavioral therapy, and wearable sensors—show promise in improving engagement and adherence (7). Closed-loop neuromodulation and minimally invasive implants could enhance therapeutic precision. Yet these innovations require robust infrastructure, clinician training, and reimbursement frameworks (4, 5).

Pharmacovigilance studies have revealed differential neuropsychiatric side effect profiles for commonly prescribed agents, emphasizing the need for more personalized prescribing. Fragmented treatment algorithms that assume linear response patterns fail to accommodate the fluctuating nature of symptoms. There is a need for adaptable, chronic care models to replace one-size-fits-all strategies.

## 5 Toward a new paradigm: multidisciplinary and personalized care

Longitudinal data from real-world studies highlight lapses in follow-up and discontinuity of care, particularly after advanced interventions (8). A chronic disease management model is essential—one that incorporates pelvic floor physical therapy, behavioral health support, shared decision-making, and ongoing patient monitoring (7).

Future care models should integrate wearable technologies, digital therapeutics, and individualized patient feedback (4, 5). Clinical trials must move beyond short-term endpoints and incorporate real-world data, patient-reported outcomes, and durability of effect. There is also a need for stratified trial design based on molecular or neurophysiologic phenotypes.

Collaboration with behavioral scientists, neurologists, and molecular biologists will expand the field’s research capacity and improve translation into clinical care. Even speculative ideas—such as epigenetic profiling or CNS-bladder interaction models—can catalyze innovation.

## 6 Conclusion

The Grand Challenge in female urology is to redefine overactive bladder as a biologically and behaviorally complex, chronically relapsing syndrome. To achieve meaningful progress, we must move beyond therapeutic recycling and invest in coordinated, multidisciplinary, and data-driven models of care. By doing so, we can overcome current limitations and build a future where patients – regardless of gender – receive accurate diagnosis, tailored interventions, and enduring relief.
